# Milk production performance of Ankole crossbreds and Holstein Friesian cattle in different production environments of Rwanda

**DOI:** 10.1007/s11250-022-03357-7

**Published:** 2022-10-22

**Authors:** Maximillian Manzi, Martin Ntawubizi, Claire d’andre Hirwa, Erling Strandberg

**Affiliations:** 1 Swedish University of Agricultural Sciences (SLU), Uppsala, Sweden; 2 Rwanda Agricultural and Animal Resources Board (RAB), Kigali, Rwanda; 3 University of Rwanda (UR), Nyagatare, Rwanda

**Keywords:** Breed groups, Milk yield, Agro-ecological zones, Rwanda

## Abstract

The aim of the study was to assess the productive performance of dairy cattle in three different agro-ecological zones of Rwanda: Congo-Nile/Western (WAZ), Central plateau/Central (CAZ), and Eastern plateau/East Agro-ecological Zones (EAZ). A single-visit multi-subject survey was conducted to obtain information on the dairy cattle performance from 51 farms. The breed groups were classified as Ankole x Holstein Friesian (AF), other Ankole crossbreds (AX), and pure Holstein Friesian (F). The F had higher (*p* < 0.001) milk yield than AF in all zones except EAZ and AF had higher (*p* < 0.0001) milk yield than AX in all zones. Across all zones, F produced 9 L more than AX and 6 L more than AF per day. Cows from EAZ had the highest average milk yield; however, it was not significantly different from CAZ. The difference that was observed between AF in EAZ and AF in the other two zones indicates that agro-ecological zones should not only be the target in livestock development activities rather additional factors such as feed availability at farm level, social economic, and market infrastructure should be considered in Rwanda.

## Introduction

The livestock sector in Rwanda accounts for 10% of the agricultural GDP and is seen as a means of reducing poverty levels in the rural countryside (NISR, 2021). Livestock production provides a stable source of milk for home consumption and sale and is seen as source of employment for numerous actors in the dairy value chain, and provides organic fertilizer for the crops and forages. The cattle population is estimated at about 1.3 million heads of which 45% are local Ankole longhorn, 33% crosses, and 22% exotic breeds (NISR, 2015). As in other tropical countries, the low productivity of indigenous cattle has made the crossbreeding a viable option to improving productivity and profit (Ojango et al., 2017). Rwanda’s projected increasing trends for human population and urbanization coupled with rising household incomes, like other countries in Sub-Saharan Africa, will lead to a substantial increase in the demand for livestock products, particularly milk and meat (FAO, 2009). In an effort to improve production, particularly milk production, the Government of Rwanda (GoR) embarked on genetic improvement programs through importation of dairy cattle and upgrading local breeds using subsidized imported and locally produced dairy cattle semen. Also, the GoR has been distributing improved cows to poor families through the GIRINKA program. Currently, many smallholder farms and some larger farms exist across the country, but the cattle breeding and on-farm performance data collection are either lacking or limited. Therefore, the current study aimed to determine the milk production performance of Ankole crossbreds and Holstein Friesian cattle under agro-ecological zones of Rwanda.

## Material and methods

### Site description

**Fig. 1:**
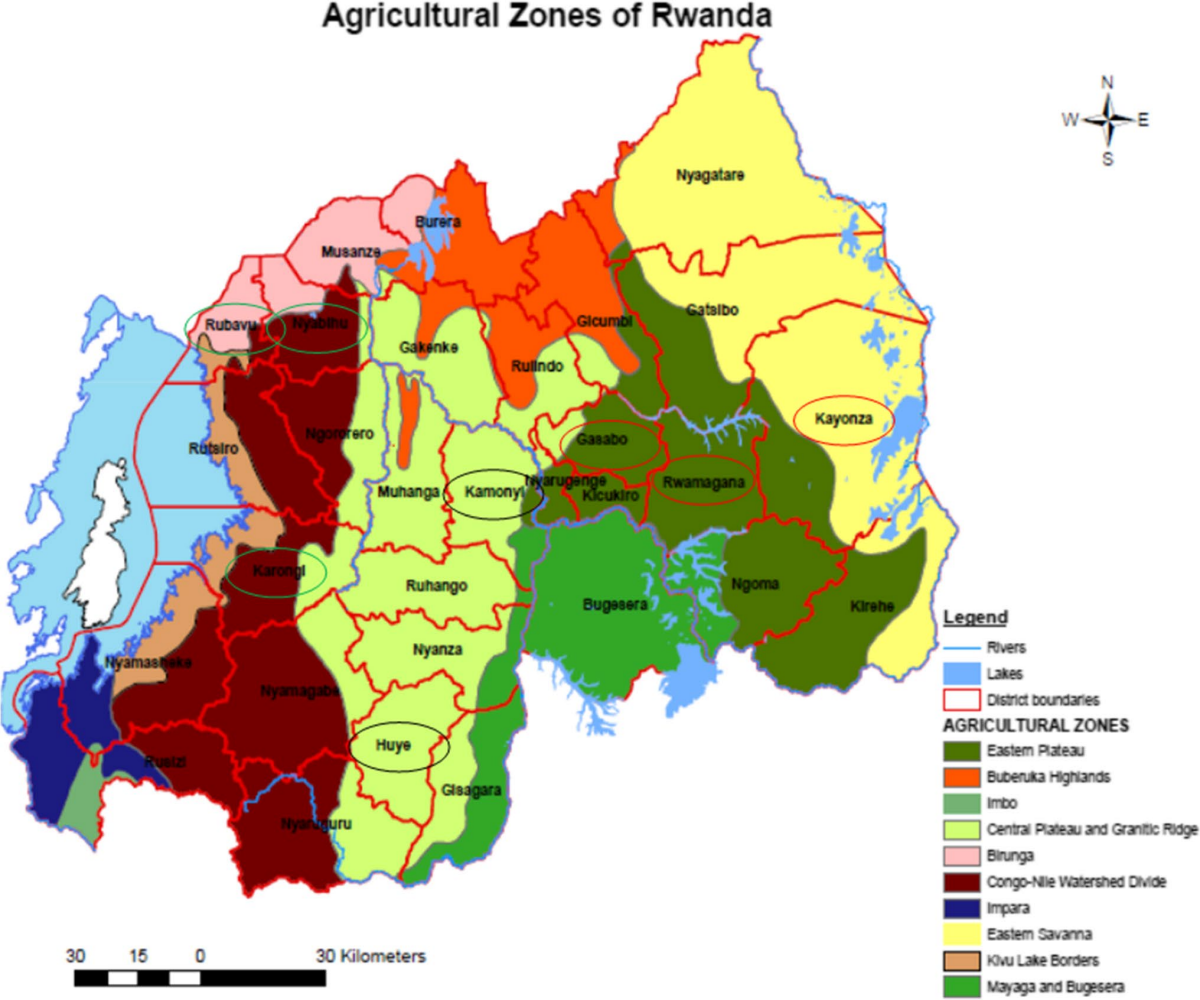
Agro-ecological zones in Rwanda (from Delepierre, G. 1975). Congo-Nile/Western Agro-ecological Zone (WAZ, green circles), Central plateau/Central Agro-ecological Zone (CAZ, black circles), and Eastern plateau/East Agro-ecological Zone (EAZ, red circles)

The study was conducted in three agro-ecological zones: Congo-Nile/Western Agro-ecological Zone (**WAZ**), Central plateau/Central Agro-ecological Zone (**CAZ**), and Eastern plateau/East Agro-ecological Zone (**EAZ**) (Fig. 1). In WAZ, Karongi, Nyabihu, and Rubavu districts were involved in the study. The zone is characterized by high altitude (1800–3000 m) and high annual rainfall (1200–1600 mm). The average annual temperature ranges between 15 and 17 °C. Livestock is dominated by crossbreds and exotic cattle and production system is mainly extensive in Nyabihu while in Karongi and Rubavu is predominantly zero grazing. Districts selected from CAZ were Huye and Kamonyi. The altitude ranges from 1100 to 1700 m, the annual rainfall varies from 1000 to 1500 mm, and the annual average temperature ranges from 18 to 20 °C. Livestock is dominated by cattle, goats, and pigs in a predominantly zero grazing practice. In EAZ, the study was conducted in the districts of Kayonza, Rwamagana, and Gasabo. In this zone, cattle and goats are the dominating livestock. Both exotic and local cattle are found in the area. The farming is characterized by mixture of zero and free grazing. The pasture is fenced by *Euphorbia* spp. In zero grazing, the cows are mainly feed on napier planted along the road, on contour hedge rows bordering planting for soil erosion control and around the marshland. In this land use system, there is variation of altitude with gentle slopes between 1200 and 1500 m above sea level. The rainfall varies between 800 and 1000 mm annually and the temperature ranges between 20 and 22 °C.

The cows were managed differently depending on feed availability under both intensive and semi-intensive management system. The feed included natural pasture (cut-and-carry), hay, milling by-products, concentrate mix, and non-conventional feeds. Cows were hand-milked twice per day. Animals were watered from piped water and mineral licks were provided ad libitum. Natural mating and artificial insemination were used for breeding cows. There was regular vaccination against notifiable diseases such as anthrax, black leg, lumpy skin disease, and foot and mouth disease. Spraying or dipping against ticks was regular and farmers called veterinarians to treat their animals whenever diseases occurred.

### Data recording and statistical analysis

This study focused on specific dairy cattle farms within different agro-ecological zones of Rwanda. Within these zones, the survey was conducted in districts known to have a large dairy cattle population. Ease of access and size of the farm were among the factors considered in purposely selecting farm and the list of farmers was acquired in collaboration with district and sector veterinary officers. The survey was conducted by University of Rwanda students as interviews in the year 2013. The respondents were either owners or managers of each dairy farm. The survey technique was of single-visit multi-subject type (ILCA, 1990). The information collected covered the following parameters: breed and age of the cow and milk yield. Milk produced at the farms was recorded for both morning and afternoon for that particular day and summed to daily milk yield. Breed groups were classified as Ankole x Holstein Friesian (AF), other Ankole crossbreds (AX, mainly Jersey, Sahiwal, and Brown Swiss), and pure Holstein Friesian (F). Combining several crosses into AX became necessary because each of these crossbreds occurred with low frequencies. Unfortunately, purebred Ankole were found almost only in CAZ and were thus not included in the analysis.

The data were entered and organized in Excel spreadsheets and statistical analyses were carried out using SAS software (SAS, 2012). The linear model included fixed effects of age of cow (in years, 2–8^+^), breed group (AF, AX, or F), agro-ecological zone (WAZ, CAZ, or EAZ), farm, and the interaction between breed group and agro-ecological zone. Differences between least squares means (LSM) were considered to be significant at the level *p* < 0.05.

## Results and discussion

Milk production during a specified period of lactation is often used as a performance indicator of dairy cows. A common measure is milk yield per lactation or per year, or average milk yield per day.

All factors in the statistical model were significant (*p* < 0.0001 except AEZ, *p* < 0.05). In total, there were 5806 cows with milk yield records and information on factors in the model. The dominant cattle breed group was pure Holstein Friesian followed by its cross with Ankole cattle (Table 1).

**Table 1:** Number of animals and least squares means (LSM) for daily milk yield (L) for three breed groups in three agro-ecological zones (AEZ)^1^

	Number of animals				LSM^2^			
	AEZ				AEZ			
Breed group^3^	WAZ	CAZ	EAZ	Total	WAZ	CAZ	EAZ	Main effect
AX	37	506	11	554	7.6^c^	2.8^a^	6.1^a^	5.5^a^
AF	363	55	870	1288	5.4 ^d^	4.6^d^	15.7^e^	8.5^b^
F	1056	660	2248	3964	11.4^f^	17.5^e^	15.8^e^	14.9^c^
Main effect	-	-	-	-	8.1^a^	8.3^ab^	12.6^b^	

^1^Congo-Nile/Western Agro-ecological Zone (WAZ), Central plateau/Central Agro-ecological Zone (CAZ), and Eastern plateau/East Agro-ecological Zone (EAZ)

^2^LSM with same superscript are not significantly different from each other (*p*-value > 0.05)

^3^*AF* Ankole x Holstein Friesian, *AX* other crossbreds with Ankole, *F* pure Holstein Friesian

The Eastern Agro-ecological Zone (EAZ) had the highest average milk yield; however, it was not significantly different from Central Agro-ecological Zone (CAZ) (Table 1). This was contrary to our expectation, because WAZ receives more annual rainfall, and hence should have a better pasture level compared to CAZ and EAZ. This indicates that there are other management factors, such as supplemental feeding, that differ between the zones.

The average milk yield varied between breed group by zone combination from 2.8 to 17.5 L (Table 1). The F had higher milk yield than AF in all agro-ecological zones except EAZ and AF had higher milk yield than AX, except in WAZ. The difference between breed groups was highest in CAZ.

In CAZ, F had 13 L higher milk yield than AF and more than 15 L higher than AX, respectively. Across all zones, F produced 9 L more than AX and 6 L more than AF per day. The difference that was observed between AF in EAZ and AF in the other two zones indicates that agro-ecological zones should not only be the target in livestock development activities. Other factors such as feed availability at farm level, socio-economic, and market infrastructure should additionally be considered. For instance, also Manzi et al. (2020) reported lower average milk yield (4.6 L) for AF in CAZ. Similarly, Nigusu and Mekasha (2016) in Ethiopia reported significant difference in the performance of Holstein Friesian and its crosses with Zebu cows in urban and secondary town dairy production systems, where a higher daily milk yield was found in the urban production systems (15.5 ± 5.2 L) and for high-grade Holstein cows (15.3 ± 4.1 L) and lower in secondary town production systems (13.7 ± 3.9 L) and for crossbred cows (11.2 ± 3.6 L). Unlike our findings, in a study analyzing livestock farming systems in Kenya, it was observed that the average daily milk production was highest in central highlands comparable to WAZ (11.8 L) followed by mid-altitude eastern region comparable to CAZ (10.4 L) and lowest in coastal lowlands comparable to EAZ (7.0 L). The reason given for increased milk production in highlands was that farmers invested in commercial concentrates for their dairy cattle (Njarui et al., 2022).

## Conclusion

The current study aimed to determine the milk production performance of Ankole crossbreds and Holstein Friesian cattle under agro-ecological zones of Rwanda. Information collected in this study indicates that milk production varied between breed groups in the three AEZ with F producing more milk on average across zones; however, there was variation between zones. Although WAZ receives more annual rainfall, and hence was expected to have better pasture level compared to EAZ, cows in EAZ had generally higher milk yield. Thus, other factors such as feed availability at farm level, socio-economic and market infrastructure should additionally be considered in Rwanda.

## Data Availability

The raw data supporting the conclusions of this article will be made available by the authors, without undue reservation.
